# Rapid quantification method of pharmaceuticals in urban wastewater using UHPLC-Orbitrap mass spectrometry—a multi-target approach for EU-directive advanced treatment evaluation

**DOI:** 10.1007/s00210-026-05480-w

**Published:** 2026-06-03

**Authors:** Uta Hofmann, Reinhard Oertel, Nancy Engel, Johannes Zander, Annemarie Lippert, Sara Schubert, Bertold Renner

**Affiliations:** 1 Institute of Clinical Pharmacology, Medical Faculty Carl Gustav Carus, TUD Dresden University of Technology, Dresden, Germany; 2 Institute of Hydrobiology, TUD Dresden University of Technology, Dresden, Germany

**Keywords:** Micropollutants, Urban Wastewater Treatment Directive (UWWTD), UHPLC-HRMS, Orbitrap mass spectrometry, German and Czech Wastewater

## Abstract

Pharmaceutical residues have become micropollutants of emerging concern in wastewater. Insufficient removal by wastewater treatment plants (WWTPs) can result in negative ecotoxicological impacts on aquatic ecosystems. Therefore, the EU Urban Wastewater Treatment Directive (UWWTD 2024/3019) requires quaternary treatment upgrades and regular monitoring in European wastewater treatment plants. This necessitates efficient and easy-to-implement wastewater analysis methods. Consequently, this study presents a novel, sensitive, and reliable HRMS-based method for the simultaneous determination of twelve indicator substances (nine UWWTD-listed and three substitutes) in WWTP influent and effluent. The method is based on solid-phase extraction (SPE) of 2 mL wastewater samples, followed by reversed-phase ultra-high performance liquid chromatography coupled to high-resolution mass spectrometry (UHPLC-HRMS) employing an Orbitrap Exploris™ 240. Detection was carried out using heated electrospray ionisation in positive ionisation mode (HESI+), except for diclofenac (HESI-). Quantification was performed using highly sensitive SIM scans, while high-resolution full-scan spectra were acquired for confirmation. Limits of quantification (LOQ) ranged mostly between 15 and 30 ng/L, except for candesartan, irbesartan, and diclofenac with LOQ > 50 ng/L. A laboratory validation demonstrated high precision and accuracy for all selected pharmaceuticals. Furthermore, the method was successfully applied to raw and treated wastewater from wastewater treatment plants (WWTPs) in the Saxon-Czech border region, which differ in terms of wastewater throughput and population equivalents. As a result, this method offers a rapid quantification approach for assessing the removal efficiency of pharmaceuticals and potential substitutes categorised in the UWWTD.

**Supplementary Information:**

The online version contains supplementary material available at 10.1007/s00210-026-05480-w.

## Introduction

Micropollutants are biologically active compounds that originate from pharmaceuticals, personal care products, pesticides, or anti-corrosion agents, which are typically present at trace concentrations (ng/L to µg/L) in environmental matrices. These substances enter the wastewater system through domestic and industrial discharges. However, at municipal wastewater treatment plants (WWTPs), they are often inadequately removed (Gurke et al. 2015a; Bhatt et al. 2022). Because of their widespread and continuous use, micropollutants are consistently released into municipal wastewater systems and ultimately discharged into aquatic environments, where they may pose risks to both aquatic ecosystems and human health (Bhatt et al. 2022; Zanni et al. 2024; Bano et al. 2025).

Therefore, the EU Urban Wastewater Treatment Directive (UWWTD 2024/3019) (The European Parliament and the Council of the European Union 2024) mandates that such substances capable of polluting water even at trace concentrations must be removed by WWTPs with a minimum efficiency of 80%. To achieve this, an additional process (quaternary treatment) must be implemented at all municipal WWTPs serving 150,000 population equivalents (PE) or more, as well as at facilities located in designated sensitive areas. All EU member states are required to implement quaternary treatment by 2045.

For the calculation of 80% pollutant removal, the EU directive specifies twelve target indicator substances (Annex 1, Part C, Para. 6), including antihypertensive drugs, one antibacterial drug, one nonsteroidal anti-inflammatory drug, one anticonvulsant, and corrosion inhibitors. These indicator compounds, based on the Swiss treatment techniques implemented in 2016, are intended to serve as proxies for the overall micropollutant load in wastewater (Van Dijk et al. 2023). They are typically ubiquitous in municipal wastewater and exhibit low removal efficiencies under conventional secondary and tertiary treatment processes (Gurke et al. 2015a; Van Dijk et al. 2023).

The indicator substances are categorised into category 1 (‘substances that can be very easily treated’) and in category 2 (‘substances that can be easily disposed of’). At least six substances from both categories must be analysed in both influent and effluent samples of the upgraded WWTPs. 

This flexible selection of only six of twelve possible indicators accounts for variability in wastewater composition and concentration levels, ensuring that suitable target analytes can be chosen depending on local conditions. In the present study, nine of the twelve listed compounds were included in the analytical method. Three compounds (hydrochlorothiazide, benzotriazole, and methyl benzotriazoles) were excluded due to analytical limitations. If less than six substances of the list can be detected in sufficient concentrations, the authorities may determine other substances to calculate the minimum removal percentage (The European Parliament and the council of the European Union 2024). For this reason, three alternatives were included, as it is shown below.

Several multi-target methods exist to detect one or more compounds from the target list in wastewater or surface water samples with high sensitivity (Alqarni 2024). These methods primarily rely on liquid chromatography coupled with mass spectrometry (LC–MS/MS or LC-HRMS), combined with solid-phase extraction (SPE) (Alqarni 2024; Ansari et al. 2025). Despite these high-tech approaches, Alqarni et al. (2024) identified several major challenges in analysing micropollutants: the wide variety of target substances, their low concentrations in samples, and matrix effects in complex sample matrices, all of which complicate method development. Additionally, analytical methods designed for high sample throughput must balance economic considerations with efficiency requirements when processing large sample volumes.

To date, no multi-target detection method exists that meets the requirements of the UWWTD and detects a sufficient number of relevant pharmaceuticals with both an adequate sensitivity and a high sample throughput. The aim of this study was to develop a method that (a) is specifically tailored to pharmaceutical contaminants (PCs) on the EU UWWTD target list, which means it includes category 1 and 2 substances as well as potential substitutes, (b) detects and quantifies the analytes in the typical concentration ranges of the influent and effluent of municipal WWTPs with high sensitivity and selectivity, and (c) is time- and cost-efficient, both in sample preparation and in the analysis step and enables a high sample throughput.

The presented method uses SPE with a low sample volume to enable a high-throughput approach. Reversed-phase ultra-high-performance liquid chromatography (UHPLC) is coupled to an Orbitrap mass spectrometer to take advantage of the superior sensitivity of the high-resolution system. It was developed as a fast single run multi-target method for the simultaneous detection of the EU-Directive mentioned PCs amisulpride (AMS), candesartan (CAN), carbamazepine (CBZ), citalopram (CIT), clarithromycin (CLR), diclofenac (DCF), irbesartan (IRB), metoprolol (MET), and venlafaxine (VEN). These are listed as standards in Table 1. Moreover, three further, not EU-mentioned surrogate compounds can be detected using the method: propranolol (PRO), tramadol (TRA), and valsartan (VAL). In addition, isotope-labelled internal standards are used to compensate for matrix effects and measurement deviations.

**Table 1:** Selected reference standards, internal standards (ISTD), and corresponding pharmacological information

Standard	CAS-Nummer	Therapeutic group	Purity (%)
Amisulpride	71675–85-9	Antipsychotic	99.73
Amisulpride-d_5_	1216626–17-3	ISTD	99.54
Candesartan	139481–59-7	Antihypertensive	96.4
Candesartan-d_5_	1189650–58-5	ISTD	> 95.0
Carbamazepine	298–46-4	Anticonvulsant	99.8
Carbamazepine-d_10_	132183–78-9	ISTD	99.2
Citalopram-HBr	59729–32-7	Antidepressant	99.0
Citalopram-d_6_-HBr	1190003–26-9	ISTD	98.87
Clarithromycin	81103–11-9	Antibacterial	99.8
Clarithromycin-d_3_	959119–22-3	ISTD	N/A
Diclofenac sodium salt	15307–79-6	Analgesic (NSAID)	99.8
Irbesartan	138402–11-6	Antihypertensive	99.9
Irbesartan-d_4_	1216883–23-6	ISTD	> 99.99
Metoprolol tartrate	56392–17-7	Antihypertensive	99.3
Metoprolol-HCl-d_7_	1219798–61-4	ISTD	98.9
Propranolol	525–66-6	Antihypertensive	N/A
Propranolol-d_7_	344298–99-3	ISTD	99.0
Tramadol-HCl	36282–47-0	Analgesic (Opioid)	99.9
cis-Tramadol-13C, d_3_-HCl		ISTD	99.33
Valsartan	137862–53-4	Antihypertensive	98.9
Valsartan-d_9_	1089736–73-1	ISTD	N/A
Venlafaxine HCl	99300–78-4	Antidepressant	N/A
Venlafaxine-HCl-d_6_	1062,606–12-5	ISTD	98.06

## Materials and method

### Chemicals

Methanol and acetonitrile, both HPLC-grade (HiPerSolv), were purchased from VWR International (Radnor, PA, USA). Ammonium acetate, disodium ethylenediaminetetraacetate (Na₂EDTA), and formic acid (conc.) were purchased from Merck AG (Darmstadt, Germany). Ultra-pure water was obtained from the in-house water treatment system GenPure, Thermo Fisher (Waltham, MA, USA). 2 mM ammonium acetate solution and formic acid (99.95/0.05, v/v) were used to prepare mobile phase solvent A, and acetonitrile and formic acid (99.975/0.025, v/v) were used to prepare solvent B, which were used for the preparation of the working solution and for the LC–MS experiments. Oasis HLB (30 mg, 1 cc) cartridges for the SPE were purchased from Waters (Manchester, UK).

Standards and internal deuterated standards (ISTD) of the pharmaceuticals were purchased from Toronto Research Chemicals, Merck AG, Cerilliant Corporation, NEOCHEMA GmbH, Wyeth, and Santa Cruz Biotechnology. Their selection was based on the microcontaminant target list of the UWWTD (see Appendix 1, Section C, Article 6, Table 3). All of the listed analytes, except for the diuretic drug hydrochlorothiazide (HCT), methyl benzotriazoles, and benzotriazole (corrosion inhibitors), are incorporated into the method presented here. PRO, VAL, and TRA have been added as possible replacement substances to calculate the minimum removal percentage. The method uses deuterated standards of the selected PCs. All substances are listed in Table 1 with corresponding information.

### Sample preparation

Validation was performed using diluted urine (1:40) from donors not taking medication containing the target analytes. The use of this surrogate was necessary due to the absence of blank wastewater, as real wastewater samples generally contain several of the analytes of interest and exhibit high variability in composition. Diluted urine provides a reproducible, matrix-containing surrogate that captures key ion suppression or enhancement effects (Rossmann et al. 2015).

To inhibit disturbing metal ions, 25 mL of sewage water was spiked with the chelating agent Na₂EDTA to a concentration of 0.8 mg/mL, shaken for 10 min, and centrifuged at 5000 × g for 10 min. Ten percent formic acid was then used for the pH adjustment (pH 3.2–3.5) of the resulting supernatant. An aliquot of 2 mL of the supernatant was taken for SPE.

### Preparation of stock solutions and quality control (QC) samples

Stock solutions of standards were prepared at concentrations of 1 mg/mL (5 mg/mL for DCF) and ISTD at 1 mg/mL in methanol and stored at − 20 °C for a maximum of 3 months. To prepare working solutions, the stocks of the standards were serially diluted (1, 0.1, 0.01, and 0.001 µg/mL; 5, 0.5, 0.05, 0.005 µg/mL for DCF) with distilled water, and the stock of ISTD was diluted to 0.1 µg/mL. All were stored at 4 °C.

Quality control samples at 0.4, 2, and 4 µg/mL (2, 10, and 20 µg/mL for DCF) were prepared in 2 mL blank urine, stored at − 20 °C, and thawed immediately before SPE. Prior to SPE, each sample was spiked with 10 µL of ISTD working solution.

### SPE

Standard (calibration curve) samples, quality control samples, and real wastewater or urine samples were all processed via SPE. Each 2 mL sample was spiked with 10 µL of ISTD working solution. SPE cartridges were then conditioned with 1 mL methanol containing 1% formic acid (v/v), followed by 1 mL of 10 mM Na₂EDTA, both applied at a constant flow rate of 10 mL/min. Two millilitre samples were loaded at 9 mL/min. Washing was performed with 1 mL ultrapure water at 10 mL/min. Elution was carried out with 0.5 mL methanol with 1% formic acid (v/v) at 10 mL/min. To prevent DCF losses, extracts were not dried but directly transferred into 2 mL glass vials (neoLab Migge GmbH, Heidelberg, Germany) for LC–MS analysis.

### LC–MS

#### UHPLC

The chromatographic separation of the target analytes was performed on a Vanquish Horizon UHPLC System from Thermo Fisher Scientific (MA, USA). All analytes were analysed using a 100 × 2.1 mm Kinetex™ Core–Shell Biphenyl column with a 1.7 µm particle size (Phenomenex, Aschaffenburg, Germany), equipped with a guard column (security guard ultra, UHPLC, Biphenyl, 5 × 2.1 mm, Phenomenex). The injection volume was 20 µL. Column temperature was held at 35 °C.

UHPLC was performed in reverse-phase mode using a binary solvent system at a flow rate of 0.4 mL/min. The separation was carried out with the following mobile-phase gradient using solvents A and B (see the ‘Chemicals’ section): 0–1 min, 95% (v/v) solvent A, and 5% (v/v) solvent B; followed by a steep linear gradient to 95% solvent B (v/v) over 4 min; held at 95% solvent B (v/v) for 1.5 min; then a steep linear gradient back to 95% solvent A (v/v) within 1.5 min; and finally, a 2.5 min hold at 95% solvent A (v/v).

#### HRMS

The UHPLC system was coupled to an Orbitrap Exploris™ 240 mass spectrometer (Thermo Fisher Scientific, San Jose, USA) using heated electrospray ionisation (HESI) operating in positive and negative ionisation mode. For spray ionisation, nitrogen was used as sheath, auxiliary, and sweep gas. The following ion-source parameters were applied: spray voltage of 3.5 kV (+) and 2.5 kV (−), ion transfer tube temperature 320 °C, vaporizer temperature 150 °C, sheath gas flow 35 arbitrary units (arb), auxiliary gas flow 7 (arb), sweep gas flow 1 (arb), and maximum spray current 10 µA.

For confirmatory purposes, analytes were verified based on retention-time matching with the isotopically labelled internal standards and accurate-mass measurement of the corresponding full-scan ions. For the MS global instrument settings, an expected peak width of 12 s was assumed. Full scans for comprehensive data recording and internal standard detection were acquired at a resolution of 90,000 (full width at half maximum, FWHM) within an m/z scan range of 100–800 (+) or 250–350 (−). SIM (single ion monitoring) scans for target detection were operated at a resolution of 60,000 (FWHM). The injection time mode was set to ‘dynamic’, with 10 desired minimum points across the peak. Retention times, time windows, and centre masses with an isolation window (m/z = 2) for SIM are listed in Table 2. AMS and MET, as well as CIT and CLR, were analysed together during SIM scans via multiplexed SIM acquisition. The data processing was carried out with Xcalibur 4.6 and FreeStyle 1.8 software (Thermo Fisher Scientific).

**Table 2:** List of pharmaceutical contaminants (PCs) and their chemical formula, HESI mode adduct (z = 1) and polarity, centre mass for SIM scans, retention time (RT) and SIM scan time window, as well as the internal (surrogate) standards used for quantification (ISTD)

PC	Formula	Adduct	Ionisation	Centre mass (m/z)	RT (min)	Window (min)	ISTD
AMS	C_17_H_27_N_3_O_4_S	+ H	Positive	370.1795	3.17	0.65	Amisulpride-d_5_
MET	C_15_H_25_O_3_N	+ H	Positive	268.1907	3.17	0.65	Metoprolol-HCl-d_5_
TRA	C_16_H_25_NO_2_	+ H	Positive	264.1958	3.24	0.50	cis-Tramadol-13C-d_3_ hydrochloride
VEN	C_17_H_27_NO_2_	+ H	Positive	278.2115	3.47	0.40	Venlafaxine-HCl-d_6_
PRO	C_16_H_21_NO_2_	+ H	Positive	260.1645	3.63	0.40	Propranolol-d_7_
CIT	C_20_H_21_FN_2_O	+ H	Positive	325.1711	3.80	0.45	Citalopram-HBR-d_6_
CLR	C_38_H_69_O_13_N	+ H	Positive	748.4842	3.80	0.45	Clarithromycin-d_3_
CBZ	C_15_H_12_N_2_O	+ H	Positive	237.1022	3.92	0.30	Carbamazepine-d_10_
CAN	C_24_H_20_N_6_O_3_	+ H	Positive	441.1670	4.09	0.30	Candesartan-d_5_
IRB	C_25_H_28_N_6_O	+ H	Positive	429.2397	4.25	0.30	Irbesartan-d_4_
VAL	C_24_H_29_N_5_O_3_	+ H	Positive	436.2346	4.34	0.30	Valsartan-d_9_
DCF	C_14_H_11_Cl_2_NO_2_	-H	Negative	294.0094	4.65	0.40	Clarithromycin-d_3_

### Method validation

#### Calibration curves and detection limits

Calibration equations were derived using a linear weighted least-squares regression analysis of the peak area ratios of analyte to ISTD versus nominal analyte concentration. For validation, six 12-point calibration curves were prepared in 2 mL blank urine, covering concentrations from 5 to 20,000 ng/L (50 to 100,000 ng/L for DCF). Linearity was assessed for these calibration curves by correlation coefficient (*R*^2^) and by determining the percentage differences between nominal and measured concentrations at each calibration point. The standard deviation (SD) of the slopes from individual calibration curves was calculated to assess between-run variability in calibration sensitivity. The relative standard deviation (RSD) was obtained by normalizing the SD to the mean slope and is expressed as a percentage, reflecting calibration reproducibility across runs.

The residual standard deviation (Sy.x) was calculated from the differences between observed and regression-predicted responses (y), where y represents the analyte-to-internal standard peak area ratio. Sy.x was defined as the square root of the sum of squared residuals divided by (*n* − 2), accounting for the two estimated regression parameters (slope and intercept). For comparability across analytes, Sy.x was additionally normalised to the mean response (ȳ) and expressed as a percentage. Sy.x was used to assess the goodness of fit and the scatter of data points around the regression line as a measure of linearity.

As a reference for the determination of the limit of detection (LOD), the Eurachem Guide (Cantwell, 2025) was followed. For each analyte, six replicate measurements of standard samples at concentration levels close to the expected LOD were used. From these measurements, the standard deviation of the signal ratios was calculated. The standard error was then determined as (1)

1 $${s}_{0}{\prime}\mathrm{=}\frac{{s}_{0}}{\sqrt{n}}$$

Here, the standard error ${s}_{0}{\prime}$ is calculated with ${s}_{0}$ as the standard deviation of the signal ratios obtained from the replicate measurements, and n is the number of replicates. Based on this, the LOD was calculated as (2)

2 $$LOD\mathrm{=}\frac{3\cdot {s}_{0}{\prime}}{\mathrm{slope}}$$

The slope was obtained from a weighted linear regression of the calibration curve constructed from low-concentration standards.

The limit of quantification (LOQ) was defined as the lowest analyte concentration for which accuracy (relative error, RE) was ≤ 20% and the signal peak height was at least three times greater than that of the blanks. The error values of RE for calibration curve standard concentrations were maximum ≤ 20% at the LOQ and ≤ 15% for other standard concentrations. Through measuring a blank sample after the highest standard, the autosampler carryover was determined.

#### Specificity

To test the specificity of the method, blank urine samples from five healthy volunteers were pretreated and analysed. The peak areas were determined for each analyte and summarised as the mean peak area to characterise the background signal of the matrix. These peak areas were compared to the peak area of standard samples at the LOQ level. For the evaluation of specificity, the mean peak areas of the blank samples were expressed as a percentage of the LOQ peak area.

#### Precision and accuracy

To evaluate precision and accuracy, three sets of spiked urine QC samples at the three concentrations described above (‘Preparation of stock solutions and quality control (QC) samples’ section) were analysed on the same day and over 3 consecutive days. For each day, samples for standard curves were prepared and analysed to calculate the concentration of QC samples. Coefficient of variation (CV, %) and relative error (AC, %) were calculated to estimate precision and accuracy.

Intra-day precision was assessed separately for each analytical run based on three replicate measurements (*n* = 3 per QC level per day) and expressed as CV. To describe variability of intra-day precision across runs, CV values obtained from 3 independent analytical days were summarised as mean CV and range (minimum–maximum). Inter-day precision was determined across three independent analytical runs (*n* = 3 days) and expressed as the CV of the mean concentrations obtained per run for each QC level.

#### Recovery

Recovery experiments were performed with three QC concentrations with three replicates each for the standards, and at one concentration for the ISTD. Analyte peak areas obtained from urine samples spiked before SPE extraction (series A) were compared to those from blank urine samples spiked with analytes after SPE extraction (i.e. from the eluate; series B). Both were adjusted to the same nominal concentrations. This approach allowed assessment of potential analyte losses during the SPE process.

#### Matrix effects

The peak areas of three different samples of urine, which were first extracted by SPE and then spiked with analytes at three QC levels (‘Recovery’ section; series B), were compared to the peak areas of corresponding standard solutions in solvent mixture A and B (series C) at the same concentrations.

The ratio (series B/series C × 100) is defined as the matrix effect. A matrix effect value of 100% indicates that the response in the mobile phase and in the urine extracts was identical, meaning no absolute matrix effect was observed. Typically, a relative standard deviation (RSD) below 10% is considered acceptable. Matrix effects for the internal standard (ISTD) were evaluated in a similar manner at one concentration.

#### Stability

The stability of the stock solutions was examined by measuring the standard concentrations after 14 days at 4 °C and after 24 h at room temperature (RT, 25 °C).

The stability of analytes in urine QC samples was tested after 24 h at RT, after three complete freeze/thaw cycles from − 20 to 25 °C, and after long-term storage at − 20 °C (14 days). Placement stability of the ready-to-inject samples was also investigated after 24 h at 10 °C in the autosampler rack. A sample was considered stable in the biological matrix when the calculated concentration was 85 to 115% of the freshly prepared samples.

### Field study—real sample analysis

For this study, seven influent and effluent samples were collected within 1 week in September 2025 from an eastern German urban wastewater treatment plant (WWTP-A) with a treatment capacity exceeding 700,000 population equivalents (PE), an annual wastewater volume of approximately 55 million m^3^, and a hydraulic retention time of 24 h. The inflow and outflow samples were flow-proportional 24-h composite samples that were automatically generated. This sampling strategy was designed to mimic the EU monitoring procedure for the determination of removal rates (RR) using the developed method.

The RRs were calculated according to a guideline for the assessment of trace organic compound elimination in municipal wastewater-treatment plants issued by the Kompetenzzentrum Spurenstoffe Baden-Württemberg (KomS, 2018), which is based on a working paper from the Ministry for the Environment, Climate and Energy of Baden-Württemberg (Germany). According to this guideline, for RR calculation, a rolling average is to be calculated based on the mean RRs of samples from the last 6 days. For each individual sampling day, the RR for every target analyte is calculated as

3 $$Removal rate=\left(1-\frac{{C}_{effluent}}{{C}_{influent}}\right)\times 100\%$$

The RR for each sampling campaign is then obtained as the mean of at least six target substances. In this study, these included amisulpride, candesartan, carbamazepine, diclofenac, irbesartan, and metoprolol. These compounds were selected because their concentrations were measured within the quantifiable range on every sampling day across the entire measurement period, with category 1 substances outnumbering category 2 substances by at least two to one. Only substances with influent concentrations at least five times higher than the limit of quantification in the inffluent were included in the calculation to ensure reliable quantification even at high removal efficiencies. Negative RRs were set to 0% in accordance with the guideline.

To further assess the overall and interregional applicability of the detection method to wastewater, samples were also analysed from five small municipal WWTPs in the Saxon-Czech border region and Northern Bohemia, Czech Republic. WWTP-B and WWTP-C are located in Saxony, Germany. WWTP-B has a treatment capacity of 17,000 PE and an annual flow of 371,000 m^3^, while WWTP-C serves 12,500 PE with an annual flow of 1,246,000 m^3^. Both plants include multiple treatment stages, such as sedimentation, chemical precipitation, and biological treatment. WWTPs D–F, located in the Ústecký and Liberecký regions of Northern Bohemia, Czech Republic, are smaller, with capacities between 600 and 8000 PE and annual flows ranging from 42,000 to 561,000 m^3^.

For the WWTPs B–E, that are smaller plants without an automatic sampling system, water samples were taken as qualitative grab samples by combining 50 mL subsamples taken at 2-min intervals over 10 min out of the flowing discharge. Sampling was performed using a custom-built manual grab sampler. Samples were transported in insulated cooling boxes and stored at 8 °C and analysed within 1–2 days. In total, PC concentrations were determined in 52 samples.

## Results and discussion

### UHPLC-HRMS

#### SPE

For analyte concentration and to prevent column contamination, solid-phase extraction (SPE) was implemented as a sample preparation step. This was particularly important because direct injections during method development caused damage to the biphenyl column, resulting in progressive retention time drift and peak broadening, despite the use of a guard column. Since the implementation of SPE, no such retention time drift or peak deterioration has been observed.

#### Chromatography

**Fig. 1:**
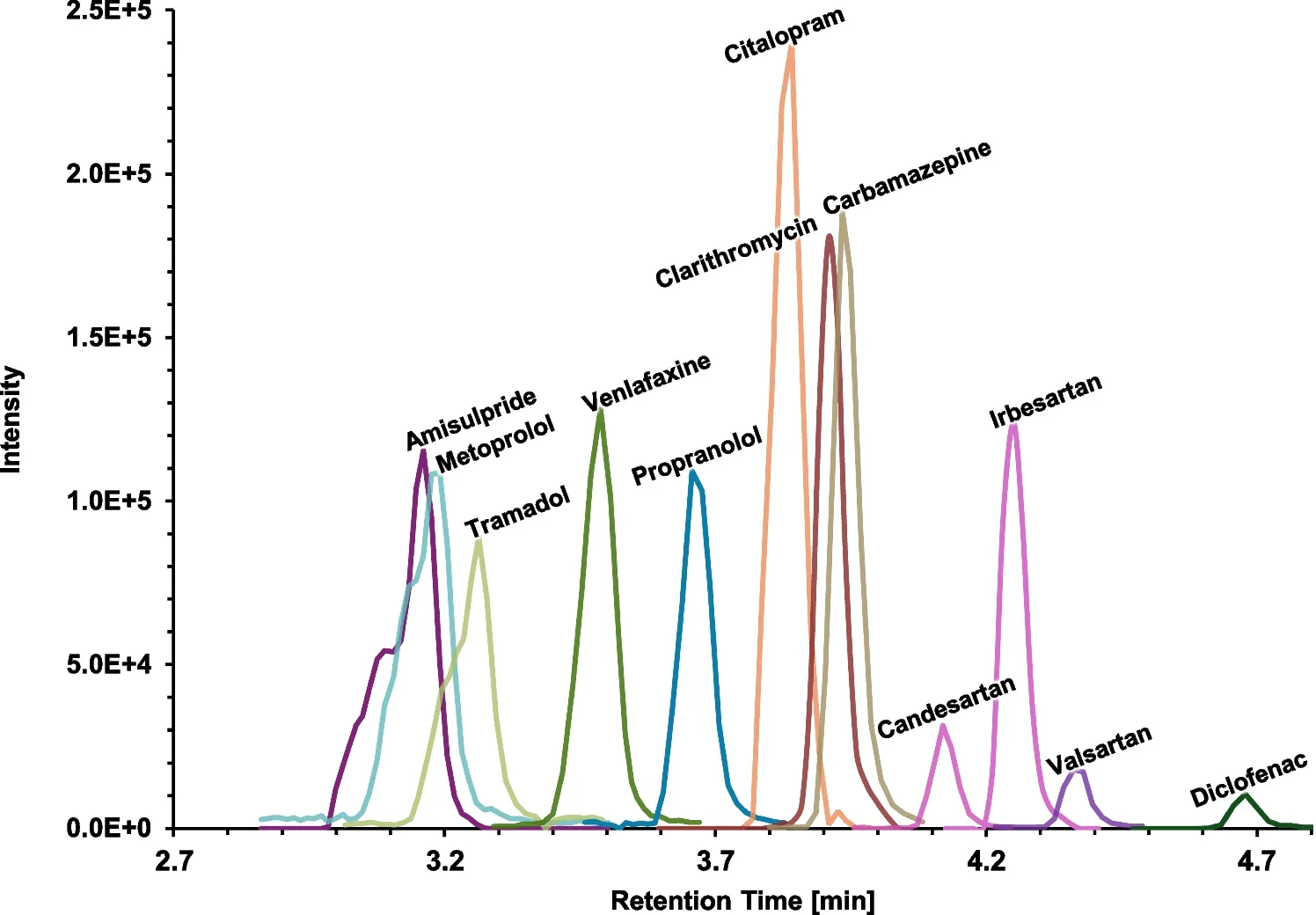
UHPLC–HRMS chromatogram (2.7–4.9 min) of blank urine spiked with pharmaceutical contaminants (1000 ng/L each; diclofenac 5000 ng/L). Analysed under HESI- and HESI+ conditions. Note: see Table 1 for chemical identities

A UHPLC biphenyl column was selected due to its suitability for the separation of aromatic analytes, enabling fast chromatographic analysis with a run time below 10 min. The chromatographic separation showed that all investigated analytes eluted between 3.0 and 4.9 min. The elution order, peak widths, and overlaps are shown in Fig. 1. Fronting was observed for AMS, MET, and TRA, due to the interaction of nonpolar methanol in the sample with the more polar mobile phase at the start of chromatography, which weakens the focusing of the analytes. However, this does not compromise the overall method performance.

CIT, CBZ, and CLR showed high signal intensities, while DCF was only detectable at about five times higher concentration levels. The low response of DCF was also observed in solvent standards, suggesting that this is not caused by matrix effects but rather by low ionization efficiency. Therefore, five times higher concentrations of DCF standards were applied in the calibration curve samples compared to the other analytes.

The UWWTD lists twelve micropollutants that can be monitored for the calculation of RRs; nine of these can be detected using the method described here. HCT, benzotriazole, and methyl benzotriazoles were not included due to analytical limitations, such as poor ionisation (HCT) or high background signals (benzotriazoles). Instead, TRA, PRO, and VAL were included in the present method, as they can be detected reliably. With these substitutions, the method still allows the selection of at least six suitable target compounds and remains compliant with the UWWTD framework.

#### MS

Resolution settings: target analyte identification was based on retention-time matching relative to the internal standard, agreement between measured and theoretical isotopic patterns, and a mass error below 5 ppm, which is considered sufficient for reliable target detection, especially in complex matrices such as wastewater (Aceña et al. 2015). High resolution is therefore crucial for the analysis. However, excessive resolution can reduce the scan rate and the number of data points per peak, potentially impairing sensitivity. Therefore, a compromise between resolution and sampling rate was applied. High-resolution full scans (90,000 FWHM at m/z = 200) and SIM scans (60,000) ensured the specificity of the method, allowing differentiation of target analytes from interfering matrix components. At these settings, mass accuracies of up to 2 ppm can be achieved (Lommen et al. 2011), providing high confidence for analyte identification based on elemental composition constraints.

When using an isotopically labelled DCF standard (+ 4 Da), an overlap with the natural isotope distribution of DCF was observed, due to the presence of molecules containing two ^37^Cl atoms, which occur at relatively high abundance (ca. 24%). This overlap compromised reliable quantification. Therefore, clarithromycin-d_3_ was used as the ISTD for DCF, which stabilised the quantification and resulted in improved validation performance compared to approaches without an internal standard or with other available deuterated standards.

##### Full scans

**Fig. 2:**
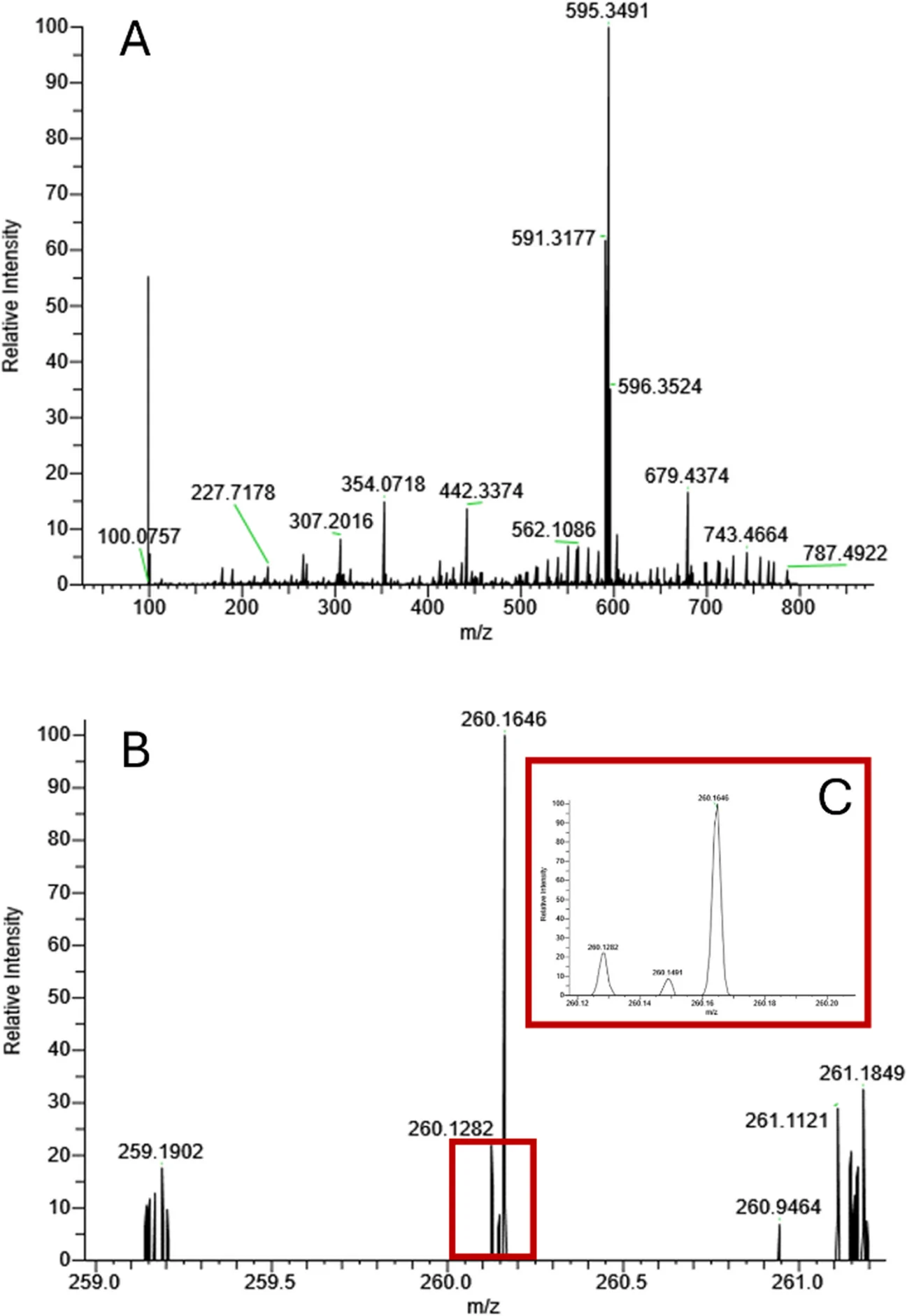
Full-scan mass spectra of a WWTP-A influent sample acquired at the retention time of propranolol (3.70 min). (**A**) Overview scan (m/z 100–800). (**B**) Zoom into m/z 259–261 showing the dominant protonated propranolol ion at m/z 260.1646. (**C**) High-resolution zoom (m/z 260.1–260.2) demonstrating separation of the target ion from co-eluting signals within a narrow mass range

There are numerous problematic substances in wastewater that are often missing from traditional monitoring lists (Hollender et al. 2019), and the same applies for the UWWTD micropollutant list. Although the EU Directive currently requires only twelve specific compounds to be monitored, the method presented here is not necessarily limited to these substances. In particular, regional and seasonal variability in consumption patterns (Gurke et al. 2015b) can justify the selection of alternative indicator substances. By using full scans in parallel with SIM scans, the method provides a comprehensive overview of sample composition within the specified m/z ranges, allowing for non-target screening, including retrospective analysis. Using full scans, the sample matrix is characterised at high m/z resolution (see Fig. 2), enabling simultaneous identification and quantification of target analytes (Krauss et al. 2010). In addition, full scans are implemented here to capture high-abundance ISTD ions.

The performance of the high mass resolution in discriminating co-eluting interferences is, for instance, illustrated in Fig. 2. A full-scan spectrum of a WWTP-A influent sample at the retention time of propranolol shows the protonated ion at m/z 260.1646, detected within a narrow mass window of ± 0.005 m/z. Despite the presence of nearby signals, the target peak is clearly resolved from co-eluting compounds. Signals differing by approximately 0.01 m/z are baseline-separated and fall outside the applied mass tolerance window.

##### SIM scans

The developed method combines high-resolution full scans with specifically placed SIM scans. The SIM scans serve to improve the detection sensitivity of low-abundance ions within an ion beam containing many other ions, by selectively monitoring target ion masses. SIM scans were established for all target analytes except DCF, as SIM mode did not improve DCF calibration curves compared with full scans.

It should be noted, however, that SIM mode efficiency decreases with an increasing number of analytes (Pugajeva et al. 2017), particularly when SIM scan windows overlap, as the measurement frequency decreases. For this reason, SIM scan time windows were set to closely match the retention times of the respective eluting analytes. When substantial overlap of SIM scan windows still occurred, the multiplexed acquisition mode (MSX) was implemented, in which multiple target ions are filtered simultaneously, thereby increasing the measurement frequency (Bourmaud et al. 2016).

##### Polarity switching

The MS setup allows polarity switching of the HESI needle within a single analytical run, but no ionisation of target analytes occurs during the time required for switching, resulting in fewer data points across chromatographic peaks (Abou-Elwafa Abdallah et al. 2019; Carlsson et al. 2022). To minimise switching, exclusively negative ionisation was applied within the DCF retention time window (~ 4.9 min), whereas only the positive ionisation mode was active during the remaining time segments.

### Method validation

#### Calibration curves and detection limits

The limits of detection (LOD), limits of quantification (LOQ), and coefficients of determination (*R*^2^) were determined in diluted blank urine to evaluate the realistic performance and typical limitations of the multi-analyte method as described above. All calibration curves were accurate and precise over wide ranges from 15 up to 15,000 ng/L. *R*^2^ exceeded 0.9963 for all analytes. The RSD of slopes was below 10% for all analytes except Diclofenac (14%), indicating generally good reproducibility of the calibration across runs. The increased variability observed for Diclofenac may be associated with limitations of the selected internal standard (see the ‘Limitations’ section). The residual standard deviation (Sy.x) was always > 0.25% for all calibration curves and averaged < 0.1% relative to the signal response. This indicates low data scatter around the regression lines and good linearity across the entire concentration range. Detailed regression data can be found in the supplementary material ESM_2 table SI-1 and SI-2.

With analysing a blank sample after the highest standard, a very low autosampler carryover (< 0.06%) could be determined (see Supplementary material ESM_2 table SI-15).

Method detection and quantitation limits were determined according to the ‘Calibration curves and detection limits’ section and are presented in Table 3. LOQs ranged from 15 ng/L (CBZ) to 325 ng/L (DCF). The LOQs are in some cases higher than those found in comparable literature (Pugajeva et al. 2017; Kachhawaha et al. 2020; Golovko et al. 2025). This is mainly due to the fact that many of these methods use larger sample volumes, such as 200 to 500 mL (Pugajeva et al.), enabling LOQs in the low ng/L to even pg/L range. Even though LOQs are influenced by many different factors, such as the instrumental setup of an LC–MS system (Golovko et al. 2025), a clear relationship can be observed between sample volume and the achievable LOQs in wastewater for pharmaceuticals as target analytes. Larger volumes result in a higher absolute analyte amount on the SPE phase and thereby enable lower LOQs.

**Table 3:** Determination of Pharmaceutical Contaminants (PCs) in blank urine with UHPLC-HRMS—validation was conducted via standard curves (*n* = 6) and linear regression. *LOD*, limit of detection; *LOQ*, limit of quantification; *ULOQ*, upper limit of quantification

Compounds	Mean LOD	Mean LOQ compound	Median ULOQ
	**[ng/L]**	**[ng/L]**	**[ng/L]**
AMS	3.1	17	20,000
CAN	5.2	63	10,000
CBZ	2.5	15	15,000
CIT	8.3	25	10,000
CLR	1.7	26	10,000
DCF	192.6	325	25,000
IRB	2.6	53	20,000
MET	9.7	28	20,000
PRO	6.1	18	7500
TRA	8.4	28	15,000
VAL	7.5	30	5000
VEN	13.8	20	20,000

In the present method, the objective was to achieve LOQs in the range of approximately 5–50 ng/L, as this range reliably covers the typical concentrations of the investigated substances in wastewater. The use of a small sample volume of 2 mL represents a compromise that, while resulting in higher LOQs compared to large-volume approaches, enables more efficient sample preparation. In particular, it reduces the time required for SPE, lowers material consumption, storage, and transport costs, and minimises laboratory space requirements.

#### Specificity

The comparison of peak areas in blank urine samples showed good specificity (< 5%) for most analytes and acceptable specificity (< 20%) for four compounds, with the highest value observed for PRO (18.7%), suggesting that this compound may be more prone to matrix-related interferences in complex environmental samples.

#### Precision and accuracy

For all analytes and across all 3 sampling days, precision ranged between 0.1 and 12.1% (intra-day) and 2.4 and 11.5% (inter-day). Higher QC concentrations generally showed lower variability, suggesting improved precision as the concentration increases. Accuracy values were between 83.0 and 115.2% (intra-day) and 90.3 and 113.5% (inter-day). With CV values below 15% and accuracy within 80–120%, the method was considered precise and accurate. The intra- and inter-day precision data for all analytes and QC levels are summarised in Table 4, which reports intra-day variability as range and mean CV across three analytical runs and inter-day variability across 3 independent days.

**Table 4:** Intra- and inter-day precision (CV) across three analytical runs. Values are based on three sets of spiked urine QC samples at three nominal concentration levels. Intra-day precision is expressed as CV (%) calculated as (standard deviation/mean) × 100 and reported as mean and range across three independent runs (*n* = 3 days). Inter-day precision was calculated across three independent runs based on mean measured concentrations per run and QC level

Compounds	Nominal conc	Intra-day CV range	Intra-day mean CV	Inter-day mean CV
	**[ng/L]**	**[%]**	**[%]**	**[%]**
Amisulpride	400	0.8–7.3	4.8	6.1
	2000	1.3–5.9	3.8	6.3
	4000	1.9–2.5	2.1	4.7
Candesartan	400	2.5–6.4	4.4	11.8
	2000	2.6–7.6	4.9	7.6
	4000	2.6–7.1	4.1	6.4
Carbamazepine	400	3.5–6.7	4.9	0.8
	2000	1.7–4.5	3.1	3.1
	4000	1.1–5.3	2.8	4.8
Citalopram	400	3.8–7.8	5.1	10.6
	2000	3–6.2	4.2	7.8
	4000	2.1–7.8	4.8	10
Clarithromycin	400	0.1–11	5.3	1.4
	2000	3.5–5.7	4.5	3.1
	4000	2–9.1	5.5	1.9
Diclofenac	2000	2–2.7	2.2	3.1
	10,000	2.9–3.3	3.1	4.3
	20,000	0.5–7.5	3.6	5.9
Irbesartan	400	2.4–4.9	4	7.3
	2000	0.6–12.1	5.8	9.3
	4000	3–9.9	6.4	8.4
Metoprolol	400	1.4–8.1	5.1	4.4
	2000	4.4–7.1	5.8	2.7
	4000	3.6–5	4.5	1.7
Propranolol	400	1.4–10.7	5.9	8
	2000	6.5–10.9	8.6	10
	4000	1.6–3	2.1	1.8
Tramadol	400	1.8–3.1	2.6	6
	2000	2.7–12	6.3	2.7
	4000	3.3–5.8	4.3	4.5
Valsartan	400	1.3–2.8	2.1	6.4
	2000	2–7.7	5.4	4.7
	4000	1–9.1	4.5	11.5
Venlafaxin	400	1.5–4	2.6	5.1
	2000	2–7.5	5.4	5.5
	4000	1–2.9	1.9	4.2

#### Recovery

**Fig. 3:**
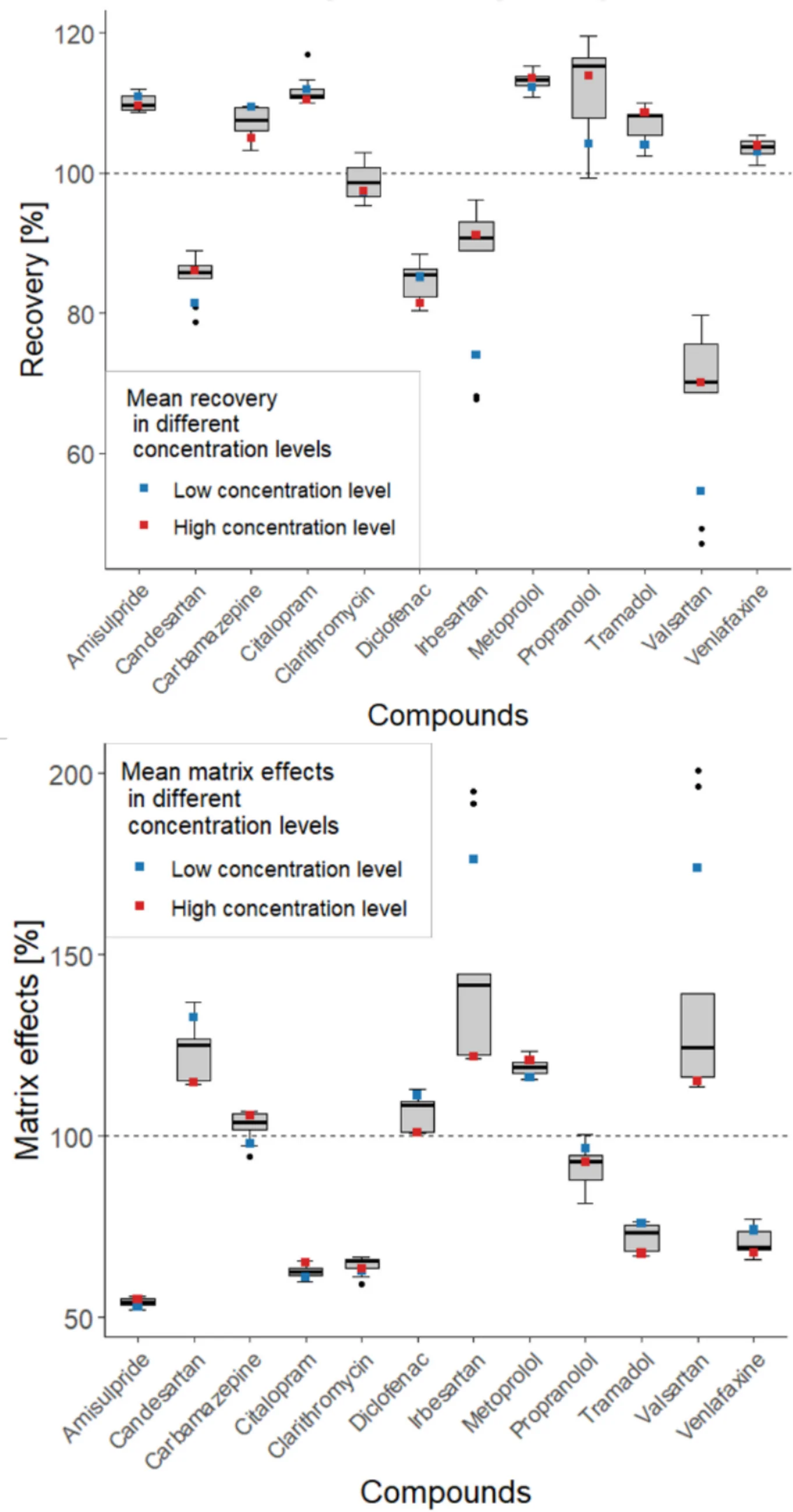
Recovery and matrix effects. Boxplots show the data distribution (*n* = 9), with whiskers indicating 1.5 × the interquartile range (IQR) and the median as the central line. Mean recoveries and mean matrix effects are shown as colored triangles for each concentration level. High concentration, 4 µg/L (20 µg/L for DCF); low concentration, 0.4 µg/L (2 µg/L for DCF)

With few exceptions, recoveries were within the acceptable 85–115% range. Typically, the observed ISTD recoveries were 5–10% higher than analyte recoveries, a range that was deemed suitable for effective recovery compensation. Substantially higher losses were observed for VAL, with a mean of 36.1% undetectable across all three concentration levels (Fig. 3). Recovery rates for each concentration level are provided in the Supplementary material ESM_2 table SI-8. Increased RSDs (> 10%) for VAL (16.4%) and IRB (12.4%) likely result from partial adsorption onto the sorbent, which has a proportionally greater effect at low concentrations. Similarly, concentration-dependent losses occurred for the corresponding ISTDs, highlighting their importance in compensating for SPE-related analyte loss or apparent signal changes.

#### Matrix effects

Substantial matrix effects were observed, including ion suppression for AMS (45%, RSD 3.4%), CIT (62.9%), CLR (64.2%), VEN (71.2%), and TRA (72.3%), and ion enhancement for CAN (124.4%), VAL (139.9%), and IRB (146.9%). In all cases, ISTDs mirrored these effects. High RSDs for IRB (18.7%) and VAL (21.8%) reflect concentration-dependent variations, with matrix effects more pronounced at 0.4 µg/L than at 2–4 µg/L (see Fig. 3 and Supplementary material table SI-7). ISTDs reliably compensate for these effects.

#### Stability

Concentration variations of the stock solutions did not exceed ± 15% after storage at 4 °C or after 24 h at room temperature (RT, 25 °C). In QC samples stored for 24 h at RT, CLR, and VAL showed concentration losses slightly exceeding 15% across all QC levels, with a maximum loss of 16.1% observed for CLR at 0.2 µg/L. Freeze–thaw cycles particularly affected DCF (− 31.3%), IRB (− 17.2%), and VAL (− 16.7%). Therefore, repeated freeze–thaw cycles, as well as prolonged storage of QC samples at RT, should be avoided. Long-term storage and storage in the autosampler for 24 h did not affect concentrations beyond the acceptable range of 85–115%.

### Field study—real sample analysis

The developed analytical method was successfully applied in a field study comprising 52 influent and effluent samples from wastewater treatment plants (WWTP-A to WWTP-F) in the Saxon-Czech border region (Northern Bohemia). The investigated substances were detected in nearly all samples, with DCF exhibiting the highest effluent concentrations (Table 5). At WWTP-A, influent concentrations of DCF ranged from 1.32 to 17.56 µg/L, while effluent concentrations ranged from 0.67 to 5.54 µg/L. These values are consistent with literature reports, including 1.9–5.2 µg/L in Berlin (Schmidt et al. 2024) and influent concentrations in the one- to two-digit µg/L range in Poland (Kołecka et al. 2022). Variability is mostly driven by regional consumption patterns and treatment processes, and occasionally seasonality (Ter Laak et al. 2014; Gurke et al. 2015b).

**Table 5:** Concentrations of pharmaceutical contaminants (PCs) in ng/L measured in 52 samples collected from six wastewater treatment plants (WWTPs A–F) in German Saxony and Czech border region over multiple sampling periods. Samples include both influent and effluent wastewater. More detailed data are provided in the Supplementary material ESM_3

**Sampling period**	***n* (samples)**	**WWTP**	**Influent/Effluent**		**AMS**	**CAN**	**CBZ**	**CIT**^**b**^	**CLR**^**b**^	**DCF**	**IRB**	**MET**	**PRO**^**c**^	**TRA**	**VAL**^**a**^	**VEN**
					**[ng/L]**	**[ng/L]**	**[ng/L]**	**[ng/L]**	**[ng/L]**	**[ng/L]**	**[ng/L]**	**[ng/L]**	**[ng/L]**	**[ng/L]**	**[ng/L]**	**[ng/L]**
04.09.2025–10.09.2025	7	A	In	Min	158	3264	616	60	< LOQ	2288	414	2132	47	345	> ULOQ	763
				Max	395	5701	1030	153	44	4228	700	3460	79	5704	> ULOQ	1281
	7		Out	Min	198	3446	710	127	46	2043	379	1791	49	841	1219	674
				Max	310	4439	844	175	75	2862	466	2322	64	> ULOQ	1458	840
11.01.2025–19.01.2025	9	B	In	Min	< LOQ	< LOQ	462	36	< LOQ	1316	< LOQ	1892	< LOQ	< LOQ	898	< LOQ
				Max	218	8772	3305	111	692	10,072	1249	5098	275	1395	> ULOQ	1021
23.12.2024–06.01.2025	15		Out	Min	75	1916	879	45	254	2406	296	1770	40	< LOQ	4132	534
				Max	227	3802	1542	83	869	5541	802	3221	99	909	> ULOQ	970
13.05.2025–01.12.2025	3	C	In	Min	551	3826	159	167	< LOQ	10,692	391	5117	116	421	>ULOQ	967
				Max	817	5721	353	248	322	13,661	1204	7603	233	703	> ULOQ	1812
	3		Out	Min	414	2710	205	161	127	4004	411	5301	132	379	14,398	984
				Max	838	4395	353	234	155	7711	518	6753	192	522	> ULOQ	1386
19.08.2025	1	D	In		35	201	2570	298	982	3883	72	1110	< LOQ	2134	80	521
13.05.2025–19.08.2025	2		Out	Min	< LOQ	103	541	178	< LOQ	674	< LOQ	683	< LOQ	516.9	617	293
				Max	860	564	2315	463	357	7476	1562	2303	24	3170	7506	743
19.08.2025	1	E	In		5665	854	1611	261	109	17,558	< LOQ	966	< LOQ	2026	10,678	625
13.05.2025–19.08.2025	2		Out	Min	3728	471	1837	198	703	2323	270	797	< LOQ	4002	268	1163
				Max	5989	593	2008	311	1426	4820	333	915	19	5019	281	1400
19.08.2025	1	F	In		261	302	839	248	262	13,855	140	1390	< LOQ	3103	12,765	672
19.08.2025	1		Out		342	270	1039	159	283	3604	231	510	< LOQ	2112	195	467

*N/A*, concentrations outside of the linear quantification range; not quantifiable. ^a^> 60% of samples above ULOQ. ^b^> 35% of samples below 5 × LOQ. ^c^> 80% of samples below 5 × LOQ

The measured high DCF concentrations are environmentally relevant, as effects on aquatic organisms have been reported at sub-µg/L to µg/L levels, with lowest observed effect concentration (LOEC) values as low as 0.01 µg/L (Acuña et al. 2015). Other compounds of concern were detected at lower concentrations but may also have ecological impacts. CBZ, frequently detected in the µg/L range in effluents (Table 5), is known for its persistence and widespread occurrence in surface waters. Reported long-term effect thresholds as low as 0.1 µg/L underline its environmental relevance even at low concentrations (Batucan et al. 2022). CLR has been linked to the promotion of antibiotic resistance in aquatic environments (Monahan et al. 2023). Antidepressants such as CIT were detected in all effluents at 0.05–0.46 µg/L, consistent with studies from Portugal, Australia, and Canada, and are known to induce behavioral and physiological effects in aquatic organisms (Liu et al. 2025).

Regarding the applicability of the developed analytical method, a mean RR of 19.6% was determined for WWTP-A under dry-weather conditions over seven consecutive sampling days, based on six suitable analytes. Calculations were performed in accordance with the EU Directive using flow-proportional 24 h composite samples. Only substances meeting the requirements of being above the LOQ, above 5 × LOQ in the effluent, and below the ULOQ were included, as it is recommended by the KomS guideline (KomS, 2018). Category 1 compounds (AMS, CBZ, DCF, MET) were taken into account relative to category 2 compounds (CDS, IRB) at a ratio of 2:1.

Nevertheless, the following limitations were considered: VAL showed high apparent RRs (≥ 90% over 7 sampling days at WWTP-A), but these could only be estimated, as influent concentrations exceeded the ULOQ of 15 µg/L in 64% of all samples (Table 5). Effluent concentrations varied considerably between wastewater treatment plants, ranging from 0.2 to > 15 µg/L in the effluent; at WWTP-A, they were approximately 1.2–1.6 µg/L, consistent with comparable studies (0.2–1.4 µg/L; Li et al. 2023). This high apparent removal of VAL is likely driven by biotransformation processes, in particular the formation of valsartan acid (Li et al. 2023). This indicates that VAL is a questionable surrogate indicator compound, although it may still be relevant for assessing environmental impacts.

Furthermore, an additional industrial discharge from a pharmaceutical manufacturer downstream of the influent sampling site of WWTP-A led to elevated TRA concentrations in the effluent (in some cases > 15,000 ng/L). Consequently, TRA had to be excluded from the RR calculation and cannot be used as an indicator compound at WWTP-A. This highlights the need for a flexible selection of suitable indicator substances.

Among the remaining compounds, DCF (28.2%) and MET (30.0%) showed the highest RRs at WWTP-A, while all other substances were lower. These overall low RRs are consistent with literature values for persistent micropollutants, as such pharmaceuticals are often insufficiently removed in conventional WWTPs (Gurke et al. 2015a; Bhatt et al. 2022). For DCF, a wide range of RRs has been reported, from negative values to nearly complete removal (Nosek and Zhao 2024). Negative RRs are commonly attributed to the reformation of conjugated metabolites or the reconversion of metabolites to the parent compound during biological treatment (Nosek and Zhao 2024) and have to be considered as zero in the calculation of RRs according to the KomS guideline. At WWTP-A, the mean RR of DCF was 28.2%, placing it in the lower to mid-range of the reported literature values. The low removal is likely driven mainly by adsorption to activated sludge and microbial transformation (Kołecka et al. 2022). Still, the hydraulic retention time of approximately 24 h at WWTP-A is generally insufficient for the complete transformation of many pharmaceuticals including DCF. This underscores the need to modernise wastewater treatment infrastructure in line with EU standards.

### Limitations

The following section outlines some methodological limitations that need to be mentioned.

Matrix effects in wastewater are difficult to predict due to high variability in matrix composition. The use of diluted urine as a surrogate matrix for method validation, as it was done here, does not fully capture the complexity of wastewater matrices and may contribute to additional uncertainty in matrix-related effects.

Furthermore, DCF was quantified without an isotopically labelled internal standard, which limited the correction of matrix effects. Although the use of a surrogate ISTD (Clarithromycin-d_3_) represents a limitation, potential quantification bias was minimised through demonstrated method performance, with intra-day precision of 2.3–4.4% CV, inter-day precision of 3.3–6.4% CV, and accuracy of 99.1–103.6%. Nevertheless, some residual bias, particularly due to differing matrix effects, cannot be excluded. Therefore, it is recommended to make use of isotopically labelled diclofenac standards with a mass shift of more than + 4 Da.

Due to the frequent lack of measurable noise in Orbitrap chromatograms, signal-to-noise-based or blank-based approaches for calculating LODs and LOQs were not practical. The LOQs were therefore defined in a pragmatic manner based on the lowest concentrations within the calibration curve, while the LODs were estimated indirectly from very low-concentration samples.

In addition, despite its general suitability as a monitoring target, VAL proved to be of limited suitability for calculating RRs in accordance with the EU directive, as concentrations exceeded the ULOQ in 33 of 52 samples. Furthermore, evidence suggests that VAL is not stable in sewage treatment plants, indicating potential degradation or transformation processes. In addition, stability tests revealed notable losses during sample storage, highlighting its limited operability for routine laboratory handling.

Calculated RRs apply only to degradation from the aqueous phase, which means they describe only the elimination from this phase or the release into this phase. The available results do not allow to determine whether the compounds are degraded/adsorbed or deconjugated/desorbed.

## Conclusion

An UHPLC-HRMS method with a short runtime (9.5 min) was developed and validated for quantifying 12 micropollutant pharmaceuticals in influent and effluent wastewater samples. The method proved to be cost- and time-efficient due to its small sample volume (2 mL), short preparation and analysis time, and polarity switching. Calibration with 11 internal standards ensured excellent linearity, precision, and accuracy.

The developed method, tailored for detecting indicator compounds under the EU-UWWTD Directive, was successfully applied in a field study, where a total of 52 influent and effluent samples from sewage treatment plants in the Saxon-Czech border area were analysed. The results showed that VAL concentrations exceeded 15,000 ng/L in over 60% of the samples, mostly in influent samples, and in addition, DFC concentrations in the µg/L range were found in most samples. Due to regional and regional concentration variabilities, it is proposed that further indicator compounds should be included in the method to compensate for such fluctuations.

Taking all this into account, the developed analytical method proved capable of generating a dataset that enabled reliable calculation of RRs in accordance with the EU directive and delivered plausible, anticipated results. The low removal rate of 19.6% at a large wastewater treatment plant (over 150,000 PE) in eastern Germany highlights the need for upgrades, such as a fourth treatment stage, to meet EU regulations and reduce environmental pollution. In addition, efforts should further focus on raising awareness among medical professionals and consumers about the responsible use of pharmaceuticals, such as topical diclofenac, to minimise the release of micropollutants into the environment.

## Supplementary Information

Below is the link to the electronic supplementary material.

ESM 1 (ZIP 214 KB)

## Data Availability

Supplementary material is provided by the following link: https://doi.org/10.25532/OPARA-1102
