# Supplementary material for: Rapid quantification method of pharmaceuticals in urban wastewater using UHPLC-Orbitrap mass spectrometry—a multi-target approach for EU-directive advanced treatment evaluation
Source: Naunyn Schmiedebergs Arch Pharmacol. 2026 Jun 3;399(11):17909–24. doi: 10.1007/s00210-026-05480-w (PMC13503438; doi:10.1007/s00210-026-05480-w)
Supplement: Supplementary file 1 [file 210_2026_5480_MOESM1_ESM.zip › ESM_1_README.pdf]

Electronic supplementary material for:

Rapid quantification method of pharmaceuticals in urban wastewater using UHPLC-Orbitrap mass spectrometry – a multi target approach for EU-directive advanced treatment evaluation

Journal: Analytical and Bioanalytical Chemistry

Uta Hofmann[a], Reinhard Oertel[a], Nancy Engel[a], Johannes Zander[b], Annemarie Lippert[a], Sara Schubert[b], Bertold Renner[a]

corresponding author: Uta Hofmann, uta.hofmann@tu-dresden.de

a) TUD Dresden University of Technology, Institute of Clinical Pharmacology, Faculty of Medicine Carl Gustav Carus, Dresden, Germany
b) TUD Dresden University of Technology, Institute of Hydrobiology, Dresden, Germany

This ZIP file contains this PDF and two additional Excel files: *ESM_2_validation.xlsx* and *ESM_3_fieldstudy.xlsx*.

ESM_2_validation.xlsx includes five worksheets summarizing the method validation results presented in Tables SI-1 to SI-15, covering calibration and regression data, inter- and intraday precision and accuracy, blank urine specificity, matrix effects and recovery, storage stability of quality control and working solutions, and carryover tests.

ESM_3_fieldstudy.xlsx comprises two worksheets: one reporting flagged micropollutant concentrations in samples from six wastewater treatment plants (WWTPs), and one providing analyte concentrations in consecutive weekly samples used to determine micropollutant removal rates according to the Urban Waste Water Treatment Directive (UWWTD).
